# Polarity Changes in the Transmembrane Domain Core of HIV-1 Vpu Inhibits Its Anti-Tetherin Activity

**DOI:** 10.1371/journal.pone.0020890

**Published:** 2011-06-02

**Authors:** Mingyu Lv, Jiawen Wang, Xiaodan Wang, Tao Zuo, Yingzi Zhu, Wei Kong, Xianghui Yu

**Affiliations:** National Engineering Laboratory For AIDS Vaccine, College of Life Science, Jilin University, Changchun, Jilin Province, People's Republic of China; University of Georgia, United States of America

## Abstract

Tetherin (BST-2/CD317) is an interferon-inducible antiviral protein that restricts the release of enveloped viruses from infected cells. The HIV-1 accessory protein Vpu can efficiently antagonize this restriction. In this study, we analyzed mutations of the transmembrane (TM) domain of Vpu, including deletions and substitutions, to delineate amino acids important for HIV-1 viral particle release and in interactions with tetherin. The mutants had similar subcellular localization patterns with that of wild-type Vpu and were functional with respect to CD4 downregulation. We showed that the hydrophobic binding surface for tetherin lies in the core of the Vpu TM domain. Three consecutive hydrophobic isoleucine residues in the middle region of the Vpu TM domain, I15, I16 and I17, were important for stabilizing the tetherin binding interface and determining its sensitivity to tetherin. Changing the polarity of the amino acids at these positions resulted in severe impairment of Vpu-induced tetherin targeting and antagonism. Taken together, these data reveal a model of specific hydrophobic interactions between Vpu and tetherin, which can be potentially targeted in the development of novel anti-HIV-1 drugs.

## Introduction

Human immunodeficiency virus type 1 (HIV-1) interacts with a series of host proteins that facilitate its replication in the cell and exploits the host cell machinery to maximize viral particle production [1]. There are multiple systems in host cells that render them resistant to viral infection through the actions of innate host cell restriction factors. Several host cell restriction factors have been identified that target specific steps in the HIV-1 lifecycle, including APOBEC3G [2], TRIM5α [3] and the recently identified tetherin protein (also known as BST-2, CD317 or HM1.24) [4], [5]. Viruses in turn have evolved to express adaptor molecules that counteract important host cell restrictions, such as illustrated by the Vif protein of HIV-1 which enhances the proteasomal degradation of APOBEC3G [6] and the Vpu protein which relieves the host restriction imposed by tetherin [4], [5].

Tetherin has been identified as an interferon-inducible antiviral host factor in HIV-1 infected cells. During the late phase of the viral replication pathway, tetherin blocks the release of nascent virions from HIV-1 infected cells at the plasma membrane and prevents viral spread [4]. It is a 28- to 36-kDa type II integral membrane glycoprotein with a unique topology which encodes a short N-terminal cytoplasmic tail, a single transmembrane (TM) spanning region, an extracellular coiled-coil domain and a putative glycosyl-phosphatidlyinositol (GPI) anchor at its C-terminus [7]. Tetherin is constitutively expressed in restrictive human cell lines, including HeLa, H9, Jurkat, Molt4, primary T lymphocytes and primary macrophages, while it is absent in cells that are permissive for particle release, such as 293T, HOS and HT1080 [4]. As expected from the formation of tethers to capture enveloped viruses, tetherin has shown broad-spectrum inhibition of the release of not only animal lentiviruses such as HIV-1 or SIV, but also other viruses such as MLV, HTLV-1, Ebola, Lassa virus and the Marburg virus [8], [9], [10]. As the name implies, tetherin is presumed to provide a physical tether between the plasma membrane and retained virions, and a recent study showed that an artificial tetherin-like protein, assembled from fragments of heterologous proteins, is able to mimic the biological activity of the native tetherin [11].

HIV-1 Vpu is a 16-kDa type I integral membrane phosphoprotein [12], [13]. It is an oligomeric protein with a short N-terminal domain, an uncleaved leader sequence that also acts as a TM domain and a longer cytoplasmic domain [14]. Two distinct biological activities have been attributed to Vpu: enhancement of viral particle release from the plasma membrane of infected cells and the specific degradation of the HIV-1 receptor molecule CD4 in the endoplasmic reticulum (ER) [15]. These two biological activities have been shown to operate via two distinct molecular mechanisms and involve two separate structural domains: the N-terminal TM domain for the enhancement of viral particle release and the C-terminal cytoplasmic domain for surface downregulation and proteasomal degradation of CD4 [16]. A conserved casein kinase II phosphorylation motif in the Vpu C-terminal cytoplasmic tail is responsible for binding to β-TrCP, which is important in mediating CD4 degradation [17]. In particular, the Vpu TM domain contains certain sequences important for the enhanced release of virus particles [18], while recent data suggest that the Vpu TM domain interacts with tetherin [19], [20].

In this study, we performed a mutagenesis study of the HIV-1 NL4-3 Vpu TM domain in an attempt to better understand its role and key region in the process of HIV-1 viral particle release and in Vpu-induced antagonism of tetherin. Most of the Vpu mutants localized to subcellular compartments in a similar pattern to that of wild-type Vpu and maintained the CD4 downregulation function. The effects of these mutants on both exogenous and endogenous tetherin were evaluated by different methods. Here, we showed that the hydrophobic binding surface for tetherin lies in the core of the Vpu TM domain. Most of the deletions in the different regions of the N-terminus of Vpu resulted in profound effects on its ability to antagonize tetherin, especially the three consecutive hydrophobic isoleucine residues in the middle region. Interestingly, two amino acid substitutions in the core region of the Vpu TM domain, Vpu TM M3IV and Vpu TM M3IT, resulted in distinct effects not only on tetherin targeting but also on Vpu-mediated enhancement of viral particle release and tetherin downregulation. The inability of the hydrophilic substitution mutant to interact with tetherin demonstrated that the structural stability of the binding surface is maintained by certain hydrophobic amino acids in the helix. Taken together, these data support a specific hydrophobic interaction model between the Vpu TM domain and tetherin, and further demonstrate the feasibility of blocking this interaction as a novel approach in anti-HIV-1 drug therapy.

## Results

### Construction and characterization of Vpu TM mutants

**Figure 1:**
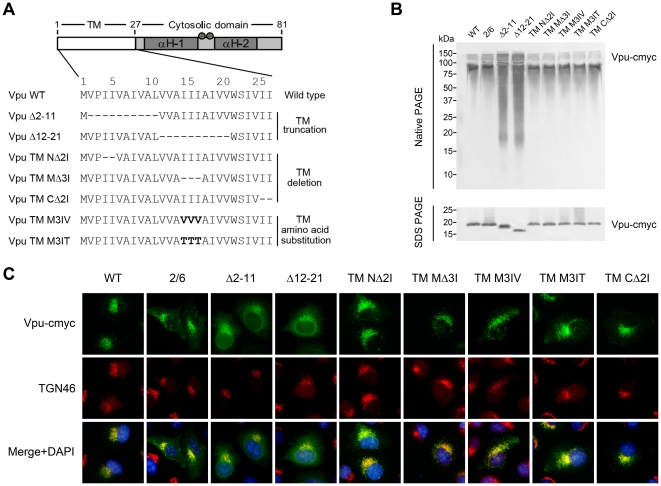
Schematic representation and basic characteristics of Vpu TM mutants. (A) Deletion and substitution mutagenesis of the Vpu TM domain. The numbering of amino acid residues used is based on the original Vpu sequence of NL4-3. The deleted amino acids are indicated by the dashes, and the substituted amino acids within the TM region are represented in bold. (B) PAGE analysis of Vpu TM mutants under native and denaturing conditions. Lysates from the Vpu variants transfected 293T cells were mixed with SDS sample buffer or native sample buffer and subjected to standard SDS-PAGE or native PAGE, respectively. The gels were transferred to a nitrocellulose membrane and further analyzed by immunoblotting using a mouse anti-myc antibody to detect myc-tagged Vpu protein. The upper panel shows the native condition and the lower panel shows denaturing condition. (C) Subcellular localization of Vpu TM mutants. HeLa cells transfected with Vpu variant expression plasmids were fixed and double stained with a mouse anti-myc antibody (green) and a rabbit anti-TGN46 antibody (red). Images were taken under a fluorescence microscope. At least 30 independent cells were examined in each sample, and the most representative cells are shown.

Previous studies have shown that the Vpu TM domain of HIV-1 is critical for virus release and that it regulates Vpu activity in the release of virus-like particles (VLPs) from the plasma membrane [18]. However, the mechanisms by which the Vpu protein enhances the release of virus particles were not clearly understood before the identification of its restriction factor tetherin. Here, we focused on analyzing conserved amino acids within the native Vpu TM domain to determine their role and relative importance in HIV-1 viral particle release and the antagonistic activities against tetherin. A series of mutations were introduced into the hydrophobic N-terminus of HIV-1 NL4-3 Vpu by PCR-directed mutagenesis, and the sequences of the mutants analyzed in this study are shown in Fig. 1A. Several deletion mutants were initially constructed, including two constructs (Vpu Δ2-11 and Vpu Δ12-21), each with a large deletion of ten amino acids in the Vpu TM domain, and three mutants with shorter deletions of two (Vpu TM NΔ2I, Vpu TM CΔ2I) or three (Vpu TM MΔ3I) conserved isoleucines in the N-terminal, middle, or C-terminal parts of Vpu TM domain. Our preliminary results based on these deletion mutants revealed that mutations in the conserved Ile motif of Vpu TM MΔ3I severely reduced its activity against tetherin. To address whether the amino acid specificity or the polarity is the more important determinant in Vpu sensitivity to tetherin, the mutants Vpu TM M3IV and Vpu TM M3IT were generated by introducing substitutions of the hydrophobic Ile residues (polarity value = 5.200) within the middle portion of the TM domain, where Ile15, Ile16 and Ile17 were replaced by Val (hydrophobic, polarity value = 5.900) or Thr (hydrophilic, polarity value = 8.600) [21]. Furthermore, a Vpu protein with both serine residues of the two casein kinase II sites changed to alanine residues (Vpu S52/56A) was used as a negative control [16]. All of the Vpu variants were expressed at approximately the same levels in HeLa and 293T cells (data not shown). Interestingly, the Vpu Δ12-21 truncated protein was obviously smaller than Vpu Δ2-11 in the SDS PAGE analysis. We hypothesized that the deletion in the Vpu Δ12-21 mutant may have generated a secretory leader peptide which was cleaved during expression, resulting in a Vpu protein devoid of most of the transmembrane domain, similar to the generation of a tetherin delTM mutant recently reported by another group [11].

Vpu is an oligomeric type I integral membrane protein that forms high-molecular-weight complexes *in vivo* and *in vitro* [14]. The oligomerization of this protein is mainly induced by its TM domain. It must remain a stable helix in this environment and oligomerize to normally interact with most other related host proteins. To verify this basic characteristic in the Vpu TM mutants, we analyzed them by native and denaturing gel electrophoresis. 293T cells transfected with Vpu variants were divided into two groups and each lysed in either RIPA buffer (denaturing) or co-immunoprecipitation (co-IP) lysis buffer (native). The cell lysates were then mixed with either SDS sample buffer or native sample buffer. The denatured samples were boiled and separated on a 12.5% SDS gel at room temperature, while the native samples remained unboiled and were separated on a 12.5% native gel in a cold room (4°C). The gels were subsequently analyzed by Western blot using an anti-myc antibody. As shown in Fig. 1B (upper panel), the native wild-type Vpu protein appeared as one major fragment with a molecular weight slightly larger than the 80 kDa reported earlier in the literature [14]. We believe that this difference in molecular weights may have been due to the additional myc tag. Among the Vpu mutants, the two truncated mutants Vpu Δ2-11 and Vpu Δ12-21 each appeared as a blurred band, the lower ends of which were similar in size to the corresponding bands on the denaturing gel. However, the other deletion and substitution Vpu TM mutants did not show obvious differences with the wild-type Vpu and Vpu S52/56A.

Although Vpu appears to act on a host protein that exerts its inhibitory activity on HIV-1 particle release at the cell surface, the most-studied subtype B Vpu was found to localize predominantly in the trans-Golgi network (TGN) and to a lesser extent in the ER and the recycling endosomes [22]. In contrast to the prototypical subtype B Vpu, however, the subtype C Vpu protein was found to localize both at the plasma membrane and in the Golgi complex [23]. The domain responsible for this TGN localization was later mapped to the Vpu TM proximal region, which contains two overlapping putative sorting signals (tyrosine-based YXXΦ and di-leucine based (D/E)XXXL(L/I)) [24]. As it is another fundamental characteristic which may affect the interaction with host proteins, the subcellular localization of the Vpu mutants were examined using immunofluorescence microscopy. Subconfluent monolayers of HeLa cells were transfected with empty vector or Vpu variant expression plasmids. Intracellular localization was evaluated by an indirect immunofluorescence technique using the anti-myc antibody to label Vpu and an anti-TGN46 antibody to stain the relevant subcellular marker as described in Materials and Methods. As shown in Fig. 1C, wild-type Vpu was mostly associated with the TGN marker and appeared as many small punctate structures which suggested that it mainly accumulated in the endosomes. Meanwhile, other Vpu mutants except for two were predominantly expressed with this similar profile. Although still partially associated with the TGN marker, the distribution pattern of Vpu Δ2-11 was moderately dispersed, while that of Vpu Δ12-21 was extensively diffuse. Except for these two truncated Vpu proteins, the patterns of the other Vpu TM mutants appeared similar to that of the wild-type Vpu and Vpu S52/56A.

From the results above, we could simply reason that the five short point mutations (Vpu TM NΔ2I, Vpu TM MΔ3I, Vpu TM CΔ2I, Vpu TM M3IV and Vpu TM M3IT) did not grossly impact the oligomerization nor the subcellular distribution of Vpu. Meanwhile, Vpu Δ2-11 and Vpu Δ12-21 failed to oligomerize, and their subcellular localizations also changed, suggesting that these truncations resulted in major damage to the TM domain. To some extent, Vpu Δ2-11 and Vpu Δ12-21 are not typical membrane Vpu proteins although they still retain several hydrophobic amino acids in the N-terminus and the Vpu C-terminal tail.

### Defective Vpu TM mutants fail to enhance HIV-1 virus release

**Figure 2:**
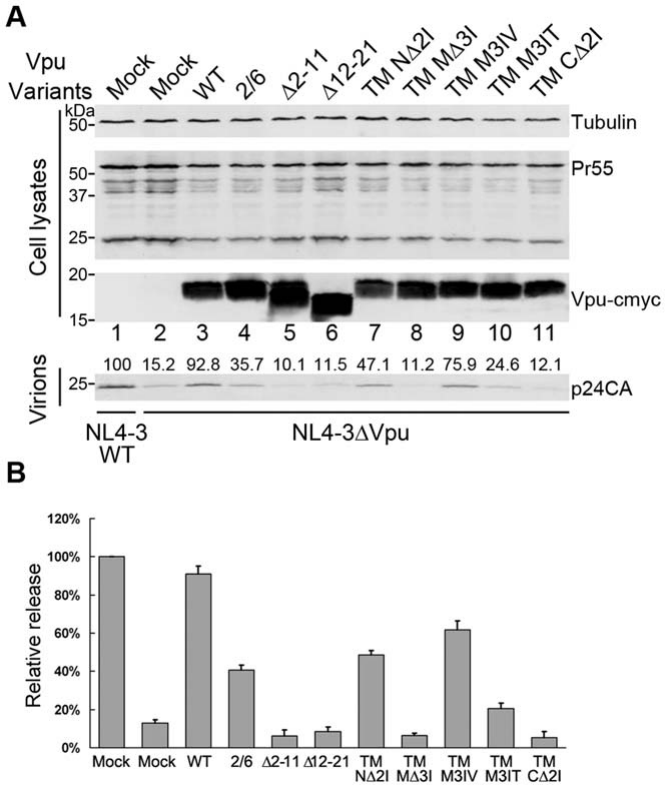
Defective Vpu TM mutants fail to enhance HIV-1 virus release. (A) VR1012 control vector or VR1012 encoding Vpu TM variants (500 ng each) was co-transfected with 1 µg proviral plasmids of pNL4-3 WT or pNL4-3ΔVpu in HeLa cells. At 48 h post-transfection, the cultured supernatants were ultracentrifuged to concentrate the virus particles. The virions and the cell lysates were analyzed by immunoblotting using an anti-p24 antibody to detect virion p24 capsid and intracellular Pr55Gag proteins, anti-myc antibody to detect myc-tagged Vpu and an antibody against tubulin to assess sample loading. The viral Pr55Gag protein was examined to exclude variations of transfection efficiency. (B) The relative infectivities of virus released from the cells transfected in (A) were assayed by infecting MAGI cells with equal volumes of supernatant samples. The cells were then fixed and stained for β-galactosidase activity. Virus release of NL4-3 WT was set to 100%. The graph was generated from two independent experiments each performed in duplicate.

Vpu is required for efficient HIV-1 particle release in certain human cells [25], and HeLa cells are a prototypic example of cells that exhibit this requirement [26]. Recent observations have shown that endogenous tetherin is constitutively expressed in HeLa cells [4]. To determine the impact of the alterations introduced into the TM domain of Vpu on its ability to enhance virus particle release, we examined the effect of these mutants on the Vpu-mediated recovery of HIV-1 Vpu defective particle release from HeLa cells. HeLa cells were co-transfected with pNL4-3 ΔVpu proviral plasmids and empty vector or Vpu variants expression plasmids. Transfection of the pNL4-3 wild-type vector along with empty vector was used as a positive control. After 48 h of incubation, HIV-1 producing cells and supernatants were harvested. Vpu was detected in the cell lysates by immunoblotting, and Pr55Gag was also detected as a transfection efficiency control. Viral particles were further isolated from the supernatants by ultracentrifugation as described in Materials and Methods. The release of viral particles was first evaluated by immunoblotting with anti-p24 capsid antibodies. As shown in Fig. 2A, the NL4-3 ΔVpu proviral clone displayed severe defects in virion p24 release compared with the NL4-3 WT clone (Fig. 2A, lanes 1 and 2), while viral p24 was recovered almost at the same level as the NL4-3 WT clone in the presence of Vpu WT (Fig. 2A, lane 3). The Vpu S52/56A retained ∼40% of the ability of the wild-type Vpu to enhance virus release (Fig. 2A, lane 4). By contrast, the two Vpu TM truncation mutants, VpuΔ2-11 and VpuΔ12-21, both exhibited significant attenuation (∼90% decrease) in the efficiency of viral particle release compared with that of the Vpu WT (Fig. 2A, lanes 5 and 6). Among the three short deletion mutants, Vpu TM NΔ2I still maintained ∼50% of the ability to enhance virus release compared to the wild-type (Fig. 2A, lane 7). Conversely, cells that expressed either the Vpu TM MΔ3I or Vpu TM CΔ2I mutant showed an impairment of ∼90% in capsid protein release (Fig. 2A, lanes 8 and 11). Importantly, cells that expressed Vpu TM M3IV released 3-fold more capsid proteins than its hydrophilic counterpart Vpu TM M3IT. This substitution mutant Vpu TM M3IV was found to recover virus release nearly as efficiently as the Vpu WT (∼75%), which suggested that the amino acid polarity at this mutation site was very important in determining the ability of Vpu to enhance virus release. To confirm these observations, we also evaluated the release of viral particles in parallel by a more sensitive method, a relative infectivity assay scored by titration of the supernatant on MAGI-CCR5 indicator cells. The amount of virus released in the supernatant was determined by comparing the ratio of relative infectious particles released in the presence of the indicated Vpu mutant to that of NL4-3 WT. As shown in Fig. 2B, the relative infectivity levels corresponded with the viral capsid protein output detected in the supernatant. From these results, we concluded that certain deletions and substitutions introduced in the TM domain of Vpu impaired its ability to optimally enhance viral particle release.

### Contribution of degradation and surface downregulation in Vpu-induced impairment of tetherin function

Although tetherin has been studied for over 3 years in the HIV-1 field, the mechanism by which Vpu antagonizes it is still not clearly defined. The explanations mainly include degradation, cell surface downregulation, and interrupting recycling of the tetherin protein. To further characterize the Vpu TM mutants using more relevant approaches, we therefore attempted to delineate these mechanisms as described below.

**Figure 3:**
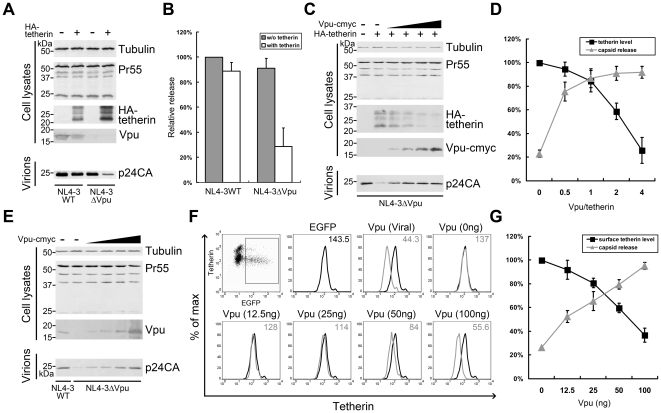
Roles of degradation and surface downregulation in Vpu-induced tetherin dysfunction. (A) 293T cells were co-transfected with 1 µg pNL4-3 WT or pNL4-3ΔVpu and 50 ng HA-tetherin or empty vector. At 48 h, the cells were examined for tetherin expression, and the pelleted virions were analyzed for p24 content. Pr55Gag was examined to exclude variations in transfection efficiencies. Tubulin was detected as a loading control. (B) The relative infectivity of virus released in (A) was assayed by infecting MAGI cells. Virus release of NL4-3 WT in the absence of tetherin was set to 100%. (C) 293T cells were co-transfected with 1 µg pNL4-3ΔVpu, 50 ng HA-tetherin and increasing doses of Vpu. HA-tetherin and Vpu-cmyc were detected in the cells. (D) The cellular tetherin levels and virus released were plotted in a line graph. The tetherin level in the absence of Vpu was set to 100%. Viral output was scored by titration of the supernatants on MAGI cells, and that without tetherin was set to 100%. (E) HeLa cells were co-transfected with 1 µg pNL4-3ΔVpu or WT, along with 500 ng pEGFP-N3, and increasing doses of Vpu. Vpu was detected in the cells with Vpu antiserum, and the pelleted virions were analyzed for p24 content. (F) Surface tetherin of cells in (E) were stained with tetherin antibodies and analyzed by flow cytometry. Cells only transfected pEGFP-N3 was used as a negative control. The samples were gated on EGFP+ cells, and surface tetherin levels are shown in histograms with median values at the top right corner. (G) The levels of surface tetherin and virus released are shown in a line graph. The tetherin level in the negative control was set to 100%. Viral output was scored by titration of the supernatants on MAGI cells, and that of NL4-3 WT was set to 100%. All values are representative of three independent experiments.

In order to investigate the relative contributions of degradation and surface downregulation to the Vpu-mediated antagonism of tetherin, we carried out the following experiments. First, we expressed HA-tetherin in Vpu-permissive 293T cells, which do not express endogenous tetherin. By measuring released capsid proteins (Fig. 3A) or by titrating the viral output (Fig. 3B), the Vpu defective HIV-1 viral particles were found to exhibit significant impairment in their ability to release from cells that express HA-tetherin, while the wild-type virus was able to overcome this inhibition. Remarkably, approximately 50% of tetherin was degraded in the presence of HIV-1 which expressed proviral Vpu. Subsequently, to delineate the correlation between Vpu-mediated tetherin degradation and dysfunction, we transfected 293T cells in the same conditions as that shown in the right panel of Fig. 3A along with increasing Vpu/tetherin ratios of expression plasmids. The results showed that the tetherin degradation was very apparent, especially in the presence of high Vpu levels, suggesting that this Vpu-mediated degradation of tetherin occurred in a dose-dependent manner (Fig. 3C). Increasing the dose of Vpu accordingly rescued the viral particle release, which paralleled the decrease in tetherin antiviral activity. However, only traces of Vpu were sufficient to efficiently suppress the tetherin antiviral function (Fig. 3D). Similar experiments were also carried out in HeLa cells, which express endogenous tetherin. HIV-1 WT was used as the positive control and pEGFP-N3 was used for confirming transfection efficiency in the FACS analysis. As shown in Fig. 3E, increasing the dose of Vpu rescued the viral particle release, and the Vpu amount seemed to correlate with the released p24 levels. By using Vpu anti-serum, Vpu-cmyc and viral Vpu could both be detected. The levels of cell surface tetherin were evaluated by flow cytometry, with cells only transfected with pEGFP-N3 and empty vector as negative controls (Fig. 3F). Samples were gated on EGFP+ cells, and the surface tetherin levels were determined and presented in the histograms with median values. As shown in Fig. 3F, HIV-1 WT which expressed viral Vpu efficiently downregulated surface tetherin, while Vpu deficient HIV-1 lost this activity. However, with increasing amounts of exogenous Vpu, the surface tetherin levels decreased in a dose-dependent manner. The amounts of Vpu, surface tetherin and capsid release levels are plotted in a line graph in Fig. 3G to better illustrate the relationships between these factors.

Taken together, these results demonstrated that surface downregulation plays an important role in the mechanism by which Vpu antagonizes tetherin. However, a poor correlation was identified between the Vpu-mediated tetherin degradation and loss of its function. Although total cellular degradation has been reported to be a redundant mechanism for the Vpu-induced tetherin antagonism in the most recent literature reports, it is indeed an important consequence of Vpu/tetherin interaction which should also be considered. To some extent, it would be of interest to compare the different profiles between endogenous and exogenous tetherin protein.

### Effects of Vpu TM mutations on Vpu-mediated degradation and surface down-regulation of tetherin

**Figure 4:**
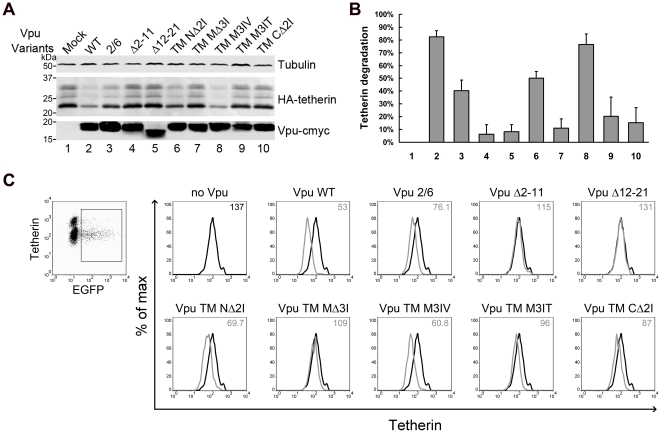
Effects of Vpu TM mutations on Vpu-mediated degradation and surface downregulation of tetherin. (A) 293T cells were co-transfected with 100 ng HA-tetherin expression plasmid along with 200 ng VR1012 control vector or VR1012 encoding Vpu TM variants at a 2∶1 molar ratio. At 48 h post-transfection, the cells were harvested for immunoblotting analysis. Tetherin and Vpu were detected with anti-HA and anti-myc antibody, respectively. Tubulin was detected as a loading control. (B) Tetherin levels were measured using Bandscan software and normalized by tubulin levels. Percentages of degraded tetherin were calculated by subtracting the densitometric intensity values of the indicated Vpu WT or mutant bands from that of the mock band to represent the different abilities of Vpu variants to mediate tetherin degradation. Values are representative of three independent experiments. (C) HeLa cells were co-transfected with 500 ng pEGFP-N3 along with 500 ng VR1012 control vector or VR1012 encoding Vpu TM variants. Cell surface tetherin was stained with BST-2 antibodies, followed by Alexa 633 goat anti-mouse IgG and analyzed by flow cytometry. Samples were gated on EGFP+ cells, and the surface tetherin levels are shown in the histograms with median values at the top right corner.

To analyze the contribution of the Vpu TM domain to the degradation of tetherin, we co-expressed HA-tetherin and Vpu variants at a fixed molar ratio that led to significant tetherin depletion, as observed in Fig. 3C. 293T cells were co-transfected with vectors expressing either the Vpu wild-type or mutated Vpu proteins and HA-tetherin. At 48 h, the cells were lysed and Vpu and tetherin were analyzed by immunoblotting. The results shown in Fig. 4A indicated that cells expressing Vpu wild-type induced a notable level of tetherin degradation, while Vpu S52/56A still retained half of the ability to induce tetherin degradation compared to the wild-type (Fig. 4A, lanes 2 and 3). Strikingly, the two Vpu TM truncation mutants, Vpu Δ2-11 and Vpu Δ12-21, exhibited strong functional defects and completely failed to mediate tetherin degradation (Fig. 4A, lanes 4 and 5). As expected, the three short deletion mutants partially affected Vpu-mediated tetherin degradation. The Vpu TM NΔ2I mutant still maintained ∼50% of the ability to degrade tetherin compared to the wild-type; however, the other two mutants Vpu TM MΔ3I and Vpu TM CΔ2I showed an extensive loss of this function. Interestingly, a couple of amino acid substitutions again exhibited distinct effects on tetherin degradation. Vpu TM M3IV was found to degrade tetherin nearly as efficiently as the Vpu WT, while the Vpu TM M3IT mutant exhibited a level of function fundamentally as defective as its corresponding deletion mutant Vpu TM MΔ3I.

To further test the ability of the Vpu variants to induce surface downregulation of endogenous tetherin, the tetherin positive HeLa cells were co-transfected with the Vpu variants and pEGFP-N3 as a marker. Cell surface tetherin was stained with anti-tetherin monoclonal antibodies and analyzed by flow cytometry. The samples were gated on EGFP+ cells, and the surface tetherin levels are shown in histograms with median values. As shown in Fig. 4C, cell surface tetherin was efficiently downregulated in cells which expressed wild-type Vpu, while the downregulation was weak in cells which expressed Vpu S52,56A. Both Vpu Δ2-11 and Vpu Δ12-21 failed to downregulate tetherin completely. The ability of Vpu TM MΔ3I to downregulate tetherin was greatly impaired, and that of Vpu TM CΔ2I was also defective to a lesser extent. Meanwhile, Vpu TM NΔ2I still retained a partial ability to downregulate tetherin. Remarkably, the substitution mutant Vpu TM M3IV could strongly downregulate tetherin, while its hydrophilic partner Vpu TM M3IT only showed slightly moderate effects in tetherin downregulation. Although the Vpu-induced tetherin antagonism was previously shown to be dependent on surface downregulation of the target protein, here it appeared that Vpu-induced cell surface tetherin downregulation assay resulted in similar profiles with the transient degradation assays. To conclude simply, the Vpu TM mutations impacted the Vpu-mediated tetherin degradation and cell surface downregulation to different extents, which essentially correlated with their capabilities to enhance virus release.

### Defective Vpu TM mutants potently block Vpu/tetherin interaction

**Figure 5:**
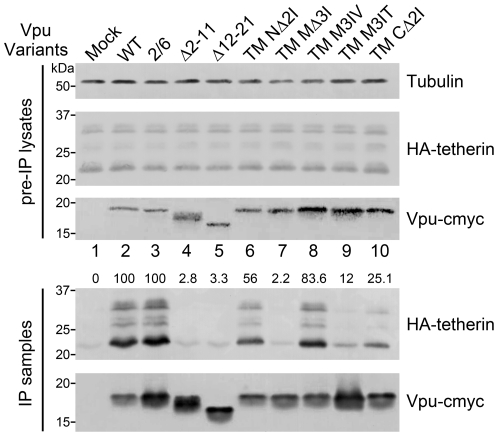
Defective Vpu TM mutants potently block the physical interaction between Vpu and tetherin. 293T cells were co-transfected with 1 µg VR1012 control vector or VR1012 encoding Vpu TM variants together with 2 µg HA-tetherin expression vector at a 1∶2 molar ratio to minimize the Vpu-mediated tetherin degradation. Cells were harvested 48 h later and subjected to immunoprecipitation using the anti-myc antibody and protein G agarose beads. Cell lysates or co-precipitated proteins were analyzed by immunoblotting to detect HA-tetherin and Vpu-cmyc. Equal loading was controlled by monitoring tubulin. The level of tetherin in each sample was quantified by densitometry, normalized by tubulin levels and shown beside the tetherin blot. The value obtained with the positive control Vpu WT was defined as 100%.

The Vpu-CD4 TM hybrid protein is known not to enhance the release of virions [18], implying that the TM domain of Vpu is critical for the interaction with tetherin. Recent studies have shown the existence of physical interactions between Vpu and tetherin [19], [27]. In order to explore the possible mechanisms involved in the enhancement of viral release and tetherin downregulation by Vpu, we tested the ability of the Vpu TM mutants to bind tetherin by co-IP. 293T cells were co-transfected with HA-tetherin in the presence or absence of Vpu variants. The Vpu mutated proteins were immunoprecipitated with anti-myc antibody, and the cell lysates and precipitates were analyzed by immunoblotting to detect the presence of tetherin. The results showed that tetherin was specifically co-precipitated with Vpu WT, and no tetherin was detected in the control precipitates in the absence of Vpu (Fig. 5, lanes 1 and 2). As expected, Vpu S52/56A also efficiently co-immunoprecipitated with tetherin. Notably, the truncated Vpu TM mutants, Vpu Δ2-11 and Vpu Δ12-21, along with the Vpu TM MΔ3I mutant with a deletion introduced in the central part of the TM domain, completely lost the ability to interact with tetherin (Fig. 5, lanes 4, 5 and 7). Densitometric measurements of the blot in Fig. 5 show that compared with the level of tetherin bound to Vpu WT (100%), ∼50% bound to Vpu TM NΔ2I, and ∼25% bound to Vpu TM CΔ2I. Remarkably, substitution mutants Vpu TM M3IV and Vpu TM M3IT displayed nearly opposite profiles of their inherent abilities to interact with tetherin (Fig. 5, lanes 8 and 9). The Vpu TM M3IV mutant, which potently down-regulated and degraded tetherin, retained >80% of its ability to interact with tetherin although less efficiently than Vpu WT. On the other hand, the Vpu TM M3IT mutant which was partially impaired in the ability to downregulate and degrade tetherin, retained only ∼10% of its tetherin-binding ability. Although this result may not be surprising, the inability of this substitution mutant to interact with tetherin demonstrated that certain hydrophobic amino acids in the Vpu TM domain may play a key role in stabilizing its tetherin binding interface and determining its sensitivity to tetherin.

### Effects of Vpu TM mutations on Vpu-mediated degradation and surface down-regulation of CD4

**Figure 6:**
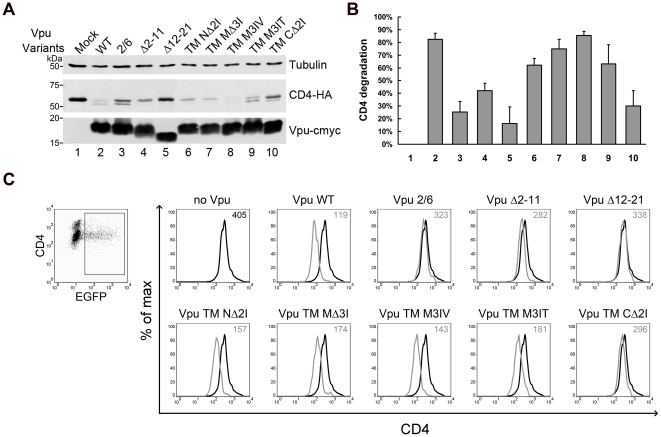
Effects of Vpu TM mutations on Vpu-mediated degradation and surface downregulation of CD4. (A) 293T cells were co-transfected with 100 ng CD4-HA expression plasmid along with 200 ng VR1012 control vector or VR1012 encoding Vpu TM variants. At 48 h post-transfection, the cells were harvested for immunoblotting analysis. CD4 and Vpu were detected with anti-HA and anti-myc antibodies, respectively. Tubulin was detected as a loading control. (B) CD4 levels were measured using Bandscan software and normalized by tubulin levels. Percentages of degraded CD4 were calculated by subtracting the densitometric intensity values of the indicated Vpu WT or mutant bands from that of the mock band to represent the different abilities of Vpu variants to mediate CD4 degradation. Results shown are the average of two independent experiments. (C) HeLa CD4 cells were co-transfected with 500 ng pEGFP-N3 along with 500 ng VR1012 control vector or VR1012 encoding Vpu TM variants. Cell surface CD4 was stained with CD4 antibodies followed by Alexa 633 goat anti-mouse IgG and analyzed by flow cytometry. Samples were gated on EGFP+ cells, and the surface CD4 levels are shown in the histograms with median values at the top right corner.

In Vpu-expressing cells, the surface CD4 receptor is downregulated and rapidly degraded in the ER [28]. The cytoplasmic domains of Vpu and CD4 are critical for Vpu-mediated down-modulation of CD4 [29], [30], although the Vpu TM domain has been implicated during this process [31]. Therefore, we also tested whether our Vpu mutants had decreased abilities to downregulate and degrade CD4. For the degradation assay, 293T cells were co-transfected with CD4-HA in the presence or absence of the Vpu variants. After 48 h, the cells were collected and analyzed by immunoblotting for CD4 levels. As expected, the wild-type Vpu caused notable degradation of CD4, while the Vpu S52/56A mutant failed to completely reduce CD4 (Fig. 6A, lanes 2 and 3). The mutants Vpu TM NΔ2I, MΔ3I, M3IV and M3IT retained the ability to degrade CD4 (Fig. 6A, lanes 6, 7, 8, 9), indicating that Vpu TM MΔ3I and M3IT are specifically defective in their interactions with tetherin. The fact that Vpu TM CΔ2I showed only a moderate reduction in its ability to degrade CD4 is also reasonable since the mutation site may be close to the cytoplasmic region (Fig. 6A, lane 10). Vpu Δ2-11 led to a moderate reduction of CD4, while that of Vpu Δ12-21, whose TM domain was nearly entirely deleted as described above, was completely blocked (Fig. 6A, lanes 4 and 5). The immunoblot of CD4 levels was also scanned and graphed to quantitate the different abilities of Vpu variants to mediate CD4 degradation (Fig. 6B).

To examine the ability of Vpu variants to downregulate surface endogenous CD4, HeLa CD4 cells were transfected with the Vpu variants and pEGFP-N3 as a marker. Cell surface CD4 was stained with CD4 antibodies and analyzed by flow cytometry. Samples were gated on EGFP+ cells, and the surface CD4 levels are shown in histograms with median values (Fig. 6C). Cell surface CD4 was significantly reduced in the presence of wild-type Vpu, while it was only slightly reduced by the expression of Vpu S52/56A. The abilities of most of the Vpu TM mutants to downregulate CD4 were not drastically affected, except for the truncations Vpu Δ2-11, Vpu Δ12-21, and Vpu TM CΔ2I whose mutation is close to the cytoplasmic region. The results of this flow cytometry analysis appeared to be consistent with the transient CD4 degradation assays. Again, Vpu had similar effects on both endogenous and exogenous CD4 proteins. However, unlike its activity against tetherin, the effects of Vpu on CD4 degradation here seemed more striking than on surface CD4 downregulation.

## Discussion

In this study, we explored the role of the HIV-1 Vpu TM domain in Vpu-mediated HIV-1 viral particle release and tetherin antagonism. We performed a mutational analysis of the Vpu TM domain in an attempt to better understand the structural requirements of this domain with respect to its biological function. On the whole, the results from our current study demonstrated that the hydrophobic binding surface for tetherin lies in the core of the Vpu TM domain. The structural stability of the binding surface is maintained by the hydrophobic amino acids in the helix, and a deletion or polarity change in this particular region of Vpu could lead to severe impairment of its tetherin antagonistic function while neither affecting its subcellular localization nor anti-CD4 function.

Research on Vpu has lasted for over 20 years since its identification [12], [13], and a large number of studies have been focused on explaining the Vpu-mediated enhancement of HIV-1 virion release. Although most earlier attempts to map the Vpu domains necessary for enhanced virus release were inconclusive, it is generally accepted that the HIV-1 Vpu TM domain is capable of forming cation-selective ion channels presumably at the plasma membrane [32], [33]. Accordingly, Paul and colleagues introduced certain mutations into the TM domain of Vpu which caused great defects in the ability of the protein to enhance the release of virus-like Gag particles [18]. It was presumed that these mutations perhaps disrupted some of the structural elements that constitute active ion channels in the membrane. In addition, many other models have been proposed over the years to delineate the mechanism of Vpu-mediated enhancement of virus release. However, the observation that fusions between Vpu-permissive and Vpu-non-permissive cells exhibit the non-permissive phenotype predicted that Vpu counteracts a host cell restriction factor for HIV-1 particle production [34]. Two groups later independently identified tetherin as the protein that specifically inhibits virion release and is counteracted by Vpu [4], [5]. Undoubtedly, the identification of this host restriction factor can provide fundamental new knowledge in the field, which has, at least temporarily, replaced earlier Vpu functional models and shifted the general focus onto the functional impairment of tetherin induced by Vpu.

Vpu localizes to the TGN and endosomal compartments, which has been shown to be necessary for its counteraction against tetherin [24]. In addition, the recycling of tetherin between the cell surface and perinuclear region is also dependent on TGN [7], [35]. Since the long truncations in Vpu Δ2-11 and Vpu Δ12-21 may have destroyed the stable helix of the TM domain in the hydrophobic environment, the gross subcellular mislocation could have resulted in the defects in tetherin antagonism. However, the diffuse localization of Vpu Δ2-11 and Vpu Δ12-21 (Fig. 1C) may because the protein is mainly in the ER (note their visible nuclear membrane). This correlates with the evidence from the native gel that suggests gross changes in Vpu oligomer structure (Fig. 1B). These observations are consistent with a quite recent study which reported that Vpu-A18H mutant was confined to an ER-like distribution, resulting in impaired downregulation of tetherin and reduced virion release [36]. Meanwhile, the other mutants that possessed small alterations in the TM domain were still able to localize in TGN (Fig. 1C). A short deletion may tighten a certain part of the transmembrane helix, while a substitution of a residue that alters the polarity may affect the electric charge distribution in a certain region. However, the observation that some of these mutations extensively impaired the tetherin antagonism of Vpu suggested that Vpu localization to the TGN and Vpu-induced counteraction against tetherin are two independent events.

Our observations of the effect of Vpu TM mutants on the enhancement of virion particle release (Fig. 2A, 2B) were essentially consistent with a previous report [18]. The Vpu TM N-terminal, middle and C-terminal deletion mutants showed moderate to profound effects in their ability to enhance viral particle release. In particular, the impairment of a Vpu TM middle deletion mutant (TM MΔ3I) was quite severe and resulted in nearly complete loss of function. These results strongly suggested that the Vpu TM domain contains determinants responsible for Vpu-mediated enhancement of viral particle release. Interestingly, the results with the mutants containing substitutions of consecutive isoleucine residues in the Vpu TM middle region hinted that the polarity of certain amino acids in this domain may greatly influence the ability of Vpu to enhance viral particle release. Although the HIV-1 Vpu contains a 27 amino acid TM domain (NL4-3 strain) at the N-terminus, it is predicted that not all of these amino acids span the plasma membrane. The observation that the Vpu TM N-terminal deletion mutants (TM NΔ2I) showed weaker impairment of tetherin antagonism than TM MΔ3I and TM CΔ2I provided evidence demonstrating that a short N-terminal tail of the TM domain may be exposed outside the phospholipid bilayers.

The precise mechanism of Vpu counteraction against tetherin to stimulate virus release is not clearly defined at present. Until now, the explanations mainly include degradation, cell surface downregulation, and interrupting recycling of the tetherin protein. Initial studies were able to measure a reduction in total cellular tetherin levels resulting from Vpu expression, and proposed a degradation-dependent mechanism [27], [37], [38], [39], which is similar to that used by HIV-1 Vif to overcome the host antiviral factor APOBEC3G [2], [6]. Notably, most of the tetherin investigated in these reports were exogenous proteins generated from transient transfection. Many studies also used flow cytometry based analyses and clearly indicated that the levels of endogenous tetherin at the cell surface of HeLa cells are markedly diminished in the presence of Vpu [5], [38], [39], [40], [41]. However, by contrast, more recent studies support a degradation-independent mechanism. Importantly, Miyagi and colleagues carried out extensive experiments in HIV-1 infected T cells and suggested that Vpu could relieve the blockade of viral release in certain cells lines in the absence of tetherin surface downregulation or depletion [40]. In addition, another group also provided evidence that Vpu can efficiently antagonize virion tethering in the absence of CD317 degradation [42]. In a very recent study, Andrew and colleagues analyzed the effects of Vpu on tetherin by performing a series of kinetic studies and indicated that surface downregulation of tetherin is caused by interference with the resupply of newly synthesized tetherin from within the cell [43], which provided a new explanation for Vpu-mediated tetherin conteraction. Finally, Douglas *et al.* has illustrated that experiments carried out in different cell types, with different tetherin sources and Vpu sources expressed at different levels, may lead to the distinct conclusions [44]. Naturally, the most reliable assay to delineate the precise antagonizing mechanism may be to investigate the interaction between viral Vpu and endogenous tetherin of host cells upon HIV-1 virus infection.

Our experiments using increasing Vpu doses were predominantly aimed at determining the reliable assay to test the effects of Vpu mutants and the contribution of degradation and cell surface downregulation in Vpu-induced impairment of tetherin function. The Vpu of HIV-1 wild-type provirus could diminish the total cellular levels of tetherin and inhibited its function (Fig. 3A, 3B), suggesting that proviral Vpu induces, at least to some extent, the degradation of exogenous tetherin. In 293T cells, Vpu provided *in trans* could rescue HIV-1 ΔVpu release and induce the degradation of tetherin in a dose-dependent manner. However, degradation of a low fraction of the total tetherin was sufficient to neutralize tetherin-mediated virion release inhibition (Fig. 3C). Meanwhile, in tetherin positive HeLa cells, increasing the dose of Vpu also rescued HIV-1 ΔVpu release and simultaneously induced the downregulation of cell surface tetherin (Fig. 3E, 3F). The negative correlations of cell surface tetherin to released virus from the HeLa cells are well-illustrated when plotted in the line graphs (Fig. 3D, 3G). Comparison of these graphs suggest that tetherin cell surface downregulation is a more significant biological consequence of and perhaps more correlative with Vpu function than tetherin degradation. Although these two assays when used to evaluate the function of Vpu mutants provided similar profiles on both endogenous and exogenous tetherin proteins (Fig. 4A, 4B, 4C), their effects on tetherin surface downregulation seemed more striking than on tetherin degradation.

There is currently controversy as to whether the β-TrCP mechanistic pathway is involved in Vpu-induced tetherin antagonism. Vpu is capable of diminishing the total amounts of tetherin in cells, and this activity relies on the recruitment of β-TrCP [27], [38], [39], [41]. The major Vpu-responsive site has now been mapped to the tetherin STS motif (positions 3 to 5) that undergoes Vpu/β-TrCP-dependent ubiquitination [45]. Nevertheless, whether Vpu antagonizes tetherin antiviral activity in a β-TrCP-dependent manner is still under debate. Here, our data demonstrated that the Vpu S52/56A mutant which was impaired for β-TrCP recruitment could perfectly interact with tetherin (Fig. 5). Moreover, Vpu S52/56A could partially enhance virion release (Fig. 2A, 2B) and still retained nearly half the capability of the wild-type Vpu to mediate tetherin cell surface downregulation (Fig. 4C) and degradation (Fig. 4A). However, as these experiments were not based on the virus infection level, we could still only presume that the β-TrCP pathway is merely involved in but does not fully account for the antagonism of tetherin by Vpu. More importantly, a recent report pointed out that β-TrCP is dispensable for the ability of Vpu to overcome the CD317/tetherin-imposed restriction on HIV-1 release [46], raising the possibility that the counteracting mechanism may involve unknown cellular factors other than β-TrCP. However, the results of our *in vivo* binding assay strongly suggested that the binding of Vpu with tetherin through the TM domain is fundamentally required for any of their complex biological interactions.

Vpu contributes to the viral effort to downregulate CD4 from the cell surface and further induce proteasomal degradation of the protein from the ER [28]. This function of Vpu begins with the recognition of the cytoplasmic tail of CD4 and recruitment of β-TrCP [47]. That Vpu S52/56A was found to be impaired in its ability to induce CD4 degradation (Fig. 6A) and surface downregulation (Fig. 6C) supported the notion that a β-TrCP-dependent mechanism is directly involved in the Vpu-induced degradation of CD4 rather than tetherin. A recent report has indicated that non-specific interactions between the hydrophobic membrane helices of CD4 and Vpu may stabilize interactions between these proteins and contribute to Vpu-induced CD4 degradation [48]. Consistent with these findings, the functional impairment of Vpu TM CΔ2I but not other TM mutants towards CD4 (Fig. 6) suggested that Vpu counteractions against both tetherin and CD4 require the Vpu TM domain, although the particular determinants may be located or overlapped at different regions of the TM domain.

While this manuscript was in preparation, Vigan and Neil [49] reported that amino acid positions A14, W22 and to a lesser extent A18 of the Vpu TM domain are required for tetherin antagonism. We acknowledge that their work presented extensive analysis of the Vpu TM domain by mutagenesis and illustrated the results by mapping the determinant amino acids in an NMR structure model created by PyMol software. They screened the Vpu TM mutants for rescue of HIV-1 ΔVpu release in 293T cells with transiently expressed tetherin and picked three of the most defective mutants to investigate the mechanism of tetherin antagonism. Although our observations are largely consistent with their study, the detailed findings are different. For example, we determined that the mutants with polarity changes at I15, I16 and I17 which severely impacted tetherin antagonism, may disturb the formation of an inside hydrophobic pocket between A14 and A18 located on the outside-facing side of the tetherin binding surface. Furthermore, we found that mutations at I26 and I27 displayed not only moderate effects of Vpu on tetherin antagonism but also a striking impact on its CD4 counteraction, providing a similar profile with W22A in the report mentioned above. Therefore, our study further strengthens the importance of the hydrophobic binding surface for tetherin antagonism which lies in the core of the Vpu TM domain. However, due to the continuity and repeatability of hydrophobic amino acids in the transmembrane helix, studies on these structures with the traditional analytical method of alanine scanning may be, at least occasionally, misleading since these membrane proteins usually do not function like enzymes or soluble adaptor proteins whose key amino acids with specific characteristics contribute greatly to their biological function. Our observation that the Vpu TM M3IV mutant could still efficiently antagonize tetherin, suggests that substitution of the region in the binding surface only with amino acids that possess similar polarity can maintain the structural integrity of the tetherin binding surface in the transmembrane helix. Meanwhile, these observations also raised the possibility that Vpu mutants with substitutions at certain amino acid positions with hydrophobic alanine may give false negative findings since they may simply mimic the structure which is potentially important for tetherin interaction. A recent report also used a similar mutation method and presented data showing that TM mutants of Vpu that cannot associate with lipid rafts also have impaired enhanced virus release function, but whether these defects are correlated with tetherin counteraction has not been determined [50]. Moreover, molecular dynamic (MD) simulations on the individual TM domains within hydrated lipid bilayers has been performed, and it was determined that the TM domain of Vpu A18H which results in impaired downregulation of tetherin and reduced virion release is incapable of interacting with the TM domain of tetherin [36]. However, obtaining the detailed structure of the Vpu TM domain in complex with tetherin by NMR or crystallography structure analysis would be most reliable method for determining the exact nature of their interaction.

Although we have not defined the exact landscape of the binding interface between Vpu and tetherin, we provided in this study new evidence suggesting that the Vpu TM domain directly contributes to the physical interaction with tetherin and additional insight into the binding model by identifying amino acids within the Vpu TM domain important for tetherin antagonism. Several NMR analyses [51], [52], [53], [54], [55] have provided us some structural features of the Vpu TM domain, and other structural studies have shed light on the structure of Vpu as it relates to its function [56], [57]. Indeed, the wealth of existing structural information has enabled the use of computational methods to clarify the mechanisms of Vpu function on an atomic scale [58], [59], [60], [61], [62]. However, a high resolution three-dimensional crystal structure of the whole Vpu protein has been challenging to obtain, predominantly because of the difficulty in producing the recombinant protein in a soluble and properly folded form.

The ultimate goal of these studies is of course to aid in the design of targeted drug therapy. The cholesterol-binding compound amphotericin B methyl ester (AME) was shown to inhibit virus release in a Vpu-dependent manner, suggesting that it may disrupt the activity of Vpu to antagonize tetherin, although the precise target site is unknown [63]. An alternative approach to disrupting Vpu function is possibly to target the Vpu-tetherin interface with small molecules or a transmembrane peptide decoy that binds the Vpu membrane-spanning domain and blocks its interactions with tetherin [64], [65]. While these inhibitors would have to interfere with a protein-protein interface, the fact that specific mutations in Vpu can block the binding of the two proteins is quite encouraging. More detailed structural analysis will be required to determine the precise mechanism of Vpu protein counteraction against tetherin during HIV-1 replication.

## Materials and Methods

### Plasmid construction

The human tetherin gene (Swiss-Prot entries Q10589) was obtained by PCR amplification from cDNA of HeLa cells and subsequently subcloned using standard molecular biology procedures into the VR1012 vector for eukaryotic expression with an HA tag added in frame at its N-terminus. The VR1012 expression plasmid encoding the codon-optimized HIV-1 NL4-3 Vpu with a cmyc tag was derived from pcDNA-Vphu [66] with primers that added a myc tag in frame at its C-terminal end. All of the Vpu mutants were engineered based on this Vpu-cmyc VR1012 clone using the QuickChange mutagenesis system (Stratagene), and sequence confirmed. The human CD4 gene (Swiss-Prot entries P01730) was obtained by PCR amplification from cDNA of H9 cells and subcloned into the VR1012 vector for eukaryotic expression with an HA tag added at its C-terminus. The HIV-1 wild-type proviral clone pNL4-3 was obtained from the National Institutes of Health AIDS Research and Reference Reagent Program (NIH-ARRRP), and the HIV-1 Vpu-defective version of the plasmid, pNL4-3 ΔVpu, was previously described [67].

### Cells and transfections

HeLa (ATCC; No. CCL-2) and HEK293T (ATCC; No. CRL-11268) cells were purchased from the American Tissue Culture Collection (ATCC), and the MAGI-CCR5 (Catalog No. 3522),HeLa CD4 (Catalog No. 459) was obtained from NIH-ARRRP. All cells were cultured in Dulbecco's Modified Eagle's Medium (DMEM) supplemented with 10% fetal bovine serum (FBS) at 37°C/5% CO_2_. Transfections of HeLa and 293T cells were performed using Lipofectamine 2000 (Invitrogen), according to the manufacturer's instructions. The MAGI-CCR5 cell line, a HeLa-CD4 cell derivative that expresses CCR5 and that has an integrated copy of the HIV-1 long terminal repeat (LTR)-driven β-D-galactosidase reporter gene, was used to analyze the relative release of the infectious HIV-1 virions.

### Immunofluorescence microscopy

Evaluation of cell-associated Vpu protein by immunofluorescence microscopy was performed as follows: HeLa cells (20–50% confluent) seeded on coverslips in a 24-well plate were transfected with 200 ng of plasmid DNA using Lipofectamine 2000. At 24 h post-transfection, cells were fixed with 2% formaldehyde in PBS for 10 min at room temperature. After fixation, cells were permeabilized with 0.25% Triton X-100 (SIGMA), blocked in 10% FBS in PBS for 20 min and then incubated with a mixture of mouse monoclonal anti-myc antibody (Millipore) and rabbit anti-TGN46 antibody (SIGMA) diluted 1∶1000 in PBS containing 1% FBS for 1 h at room temperature. Cells were washed three times in PBS and stained with a mixture of Alexa Fluor 488 conjugated goat anti-mouse IgG (Molecular Probes, Invitrogen) and Rhodamine Red-X goat anti-rabbit IgG (Molecular Probes, Invitrogen) diluted 1∶1000 in 1% FBS in PBS for 1 h at room temperature. Stained cells were washed three times in PBS. The coverslips were mounted on glass slides with mounting medium cotaining DAPI (Invitrogen). The samples were observed on an Olympus IX71 fluorescence microscope, and photos were taken with a Nikon CCD camera and associated imaging system.

### Flow cytometry analysis

HeLa cells plated in 6-well plates were transfected with indicated expression plasmids along with an EGFP-expressing vector pEGFP-N3 (Clontech) as a transfection marker. After 48 h, the cells were washed twice with PBS and non-specific binding sites were blocked for 10 min with 10% FBS in PBS. Cell surface tetherin was stained with an anti-BST-2 monoclonal IgG1 antibody (Abnova) followed by Alexa Fluor 633 conjugated goat anti-mouse IgG (Molecular Probes, Invitrogen) and analyzed on a MoFlo XDP cell sorter (Beckman Coulter). The data was then group analyzed with FlowJo 7.6.2 (Tree Star). Samples were gated on EGFP+ cells, and the surface tetherin levels are shown in histograms with median values on the top right corner. A similar assay was used to measure the cell surface CD4 levels in HeLa CD4 cells, using an anti-CD4 monoclonal antibody (NIH-ARRRP) as the primary antibody.

### Protein analysis

Unless otherwise indicated, the cells were lysed with RIPA buffer 48 h post-transfection. Cell lysates from transfected cells were mixed with SDS sample buffer and boiled for 5 min. The samples were subjected to standard SDS-PAGE and then transferred to a nitrocellulose membrane for Western blotting. Overexpressed tetherin was detected with a mouse monoclonal anti-HA antibody (Covance) against the HA-tag added on its N-terminus. While the relative individual tetherin bands in the 28–37 kDa range varied, the predominant band was analyzed by densitometric scanning. Overexpressed Vpu was detected with a mouse monoclonal anti-myc antibody (Millipore) against the myc-tag added on its C-terminus. Viral Vpu was detected with rabbit polyclonal anti-Vpu serum (NIH-ARRRP). Pr55Gag and p24CA were detected with a monoclonal anti-HIV capsid (p24) antibody obtained from HIV-1 p24 hybridoma (NIH-ARRRP) cultured supernatant. Tubulin was detected with a mouse monoclonal anti-tubulin antibody (Covance). Alkaline phosphatase conjugated goat anti-mouse and goat anti-rabbit IgG (Jackson Immunoresearch) were used as secondary antibodies for Western blotting. For the native PAGE, cells were lysed with 100 µl co-IP lysis buffer containing 50 mM Tris-HCl (pH 7.4), 150 mM NaCl, 1% Triton X-100 (SIGMA), 1% CHAPS (SIGMA) and a protease inhibitor cocktail (Roche). The lysates were then mixed with native sample buffer without SDS. The samples remained unboiled and separated on a native gel without SDS in a cold room.

### Virion production and infectivity assay

HIV-1 particles were produced by transient transfection of HeLa or 293T cells in a 6-well plate with 1 µg proviral constructs and indicated amount of other plasmids. Two days later, 2 ml supernatants of producer cells were harvested, clarified by centrifugation and passed through a 0.22 µm filter. The viral particles were pelleted through a 20% sucrose layer at 110,000× *g* for 1 h and resuspended in 30 µl RIPA Buffer. Virus particle pellets and corresponding cell lysates were analyzed by SDS–PAGE and Western blot assays using an anti-p24 capsid antibody. In single-cycle infectivity assays, 100 µl of the filtered supernatant were mixed with DEAE-dextran (SIGMA) at a final concentration of 15 µg/ml and incubated with MAGI-CCR5 indicator cells for 1 h, followed by addition of 500 µl fresh medium. At 48 h after infection, the infected cells were fixed, stained for β-galactosidase activity and analyzed by counting the blue dots representing released virion yield.

### Co-immunoprecipitation

Transfected 293T cells were lysed on ice for 30 min in 500 µl lysis buffer containing 50 mM Tris-HCl (pH 7.4), 150 mM NaCl, 1% Triton X-100 (SIGMA), 1% CHAPS (SIGMA) and a protease inhibitor cocktail (Roche), followed by centrifugation at 10,000× *g* for 10 min at 4°C to pellet the cellular debris. The clarified cell lysates were pre-cleared with 30 µl of a 25% Protein G Agarose slurry (Roche) rocking at 4°C for 1 h, and then the agarose beads were removed by centrifugation at 1000× *g* for 5 min at 4°C. The immunoprecipitations were performed on the pre-cleared supernatants by adding 1 µg of mouse anti-myc antibody for 1 h at 4°C, followed by adding 30 µl of the 25% Protein G agarose slurry and incubating for 2 h at 4°C. The beads were centrifuged at 1000× *g* for 5 min at 4°C and washed with 500 µl lysis buffer. The centrifugation and wash steps were repeated three times. The supernatant was removed after the final centrifugation, and the pellet was resuspended in 30 µl glycine HCl pH 2.0 elution buffer. The eluates were mixed with SDS-PAGE loading buffer, separated by SDS-PAGE and analyzed by immunoblotting. After being transferred onto nitrocellulose membrane, HA-tetherin and Vpu-cmyc were detected in the Western blots using anti-HA (1∶2000 dilution) and anti-myc (1∶2000 dilution) antibodies, respectively.
